# Electroacupuncture alleviates paclitaxel-induced peripheral neuropathy by reducing CCL2-mediated macrophage infiltration in sensory ganglia and sciatic nerve

**DOI:** 10.1186/s13020-024-01023-8

**Published:** 2025-01-13

**Authors:** Yuanyuan Li, Ruoyao Xu, Muyan Chen, Kaige Zheng, Huimin Nie, Chengyu Yin, Boyu Liu, Yan Tai, Junying Du, Jie Wang, Jianqiao Fang, Boyi Liu

**Affiliations:** 1 Department of Neurobiology and Acupuncture Research, The Third Clinical Medical College, Key Laboratory of Acupuncture and Neurology of Zhejiang Province, Zhejiang Chinese Medical University, Hangzhou, China; 2 Academy of Chinese Medical Sciences, Zhejiang Chinese Medical University, Hangzhou, China; 3 Department of Rehabilitation in Traditional Chinese Medicine, The Second Affiliated Hospital of Zhejiang University School of Medicine, Hangzhou, China

**Keywords:** Paclitaxel, Chemotherapy, Neuropathy, Macrophages, Acupuncture, Pain

## Abstract

**Background:**

Paclitaxel-induced peripheral neuropathy (PIPN) is prevalent among patients receiving paclitaxel chemotherapy, which results in sensory abnormality as well as neuropathic pain. Conventional medications lack effectiveness on PIPN. Clinical trials identified beneficial effects of acupuncture on PIPN among patients receiving chemotherapy. Here we explored the mechanisms underlying how acupuncture might alleviate PIPN.

**Methods:**

A mouse model of PIPN was established by repeated paclitaxel application. Electroacupuncture (EA) was applied at ST36 and BL60 acupoints of model mice. Immunostaining, flow cytometry, behavioral assay, in vivo imaging were utilized for effects determination and mechanism exploration.

**Results:**

EA ameliorated mechanical and cold pain hypersensitivities, reduced sensory neuron damage and improved loss in intra-epidermal nerve fibers (IENFs) in model mice. Macrophages infiltration were detected in DRG and sciatic nerve of model mice, which was reduced by EA. EA affected M1-like pro-inflammatory macrophage infiltration in DRG, whereas it did not affect M2-like macrophages. DRG neurons released chemoattractant CCL2 that recruited macrophages via CCR2 to DRG. EA reduced CCL2 overproduction by DRG neurons and reduced macrophage infiltration. Blocking CCR2 mimicked EA’s anti-allodynic effect, whereas exogenously applying recombinant CCL2 reversed the ameliorative effect of EA on macrophage infiltration and abolished EA’s anti-allodynia on model mice. EA ameliorated other signs of PIPN, including sensory neuron damage, sciatic nerve morphology impairment and IENFs loss. In mice inoculated with breast cancer cells, EA didn’t affect paclitaxel-induced antitumor effect.

**Conclusions:**

These findings suggest EA alleviates PIPN by reducing CCL2/CCR2 mediated-pro-inflammatory macrophage infiltration into sensory ganglia as well as the sciatic nerve. Our study supports EA could be used as a potential non-pharmacological therapy for PIPN.

**Supplementary Information:**

The online version contains supplementary material available at 10.1186/s13020-024-01023-8.

## Introduction

Paclitaxel is commonly utilized for treating many types of solid tumors, which include breast, ovarian and lung cancer [1]. Chemotherapy with paclitaxel can result in peripheral neuropathy in the extremities of the patients, a term called chemotherapy-induced peripheral neuropathy (CIPN), which represents an obvious adverse effects of chemotherapy with paclitaxel [1, 2]. Paclitaxel-induced peripheral neuropathy (PIPN) triggers neuropathic pain-like symptom that is featured with on-going pain, numbness as well as mechanical and cold hypersensitivities in the patient [3, 4]. These sensory abnormalities can last from months to even years and markedly impaired the life of the patient receiving chemotherapy with paclitaxel [5]. Till now, the antidepressant duloxetine is the only drug that has appropriate evidence to support the use for patients with established painful CIPN [6]. Nonetheless, the amount of benefit from duloxetine is moderate and it further may produce certain adverse effects [6–8]. Hence, it is an urgent necessity to identify novel treatment options for CIPN.

There is a growing number of evidence that demonstrates a critical contribution of macrophages to initiating and maintaining neuropathic pain via neuro-immune crosstalk [9, 10]. During neuronal injuries, the injured neurons and resident macrophages can release certain pro-inflammatory mediators that further induce macrophage infiltrations into the peripheral sensory system [9]. The infiltrated macrophages then release large amounts of inflammatory cytokines or mediators that can cause neuroinflammation, peripheral sensitization and chronic pain [11, 12]. It is already observed that there is remarkable macrophage infiltration in dorsal root ganglion (DRG) and peripheral sensory nerve in PIPN model animals [13, 14]. Depleting macrophages significantly relieved pain hypersensitivities, as well as neuronal apoptosis and a loss in the intra-epidermal nerve fiber (IENF) of model rats, demonstrating a crucial contribution of the infiltrated macrophages to the etiology of PIPN [13, 15]. The potent macrophage chemoattractant CCL2 has been found to be significantly increased in DRG neurons of PIPN model rats [13]. CCL2 activates CCR2 expressed on macrophages to produce macrophage chemotaxis [16]. Neutralization of CCL2 ameliorates macrophage infiltration in DRG and reduces pain in PIPN model rats [13]. *Ccr2* gene knockdown also alleviates pain in PIPN model rats [17]. These findings indicate a critical contribution of CCL2/CCR2 signaling in DRG to macrophage infiltration and PIPN etiology.

Acupuncture has long been used to ameliorate many types of chronic pain conditions, with well tolerance and minimum side effects [18, 19]. Clinical studies have demonstrated the application of acupuncture to improve peripheral neuropathy conditions in patients with diabetes or CIPN [20, 21]. Recently, a number of random clinical trials have confirmed the therapeutic actions of acupuncture on pain in patients undergoing chemotherapy using paclitaxel, with well tolerance and safety [22, 23]. The current understanding of mechanisms underlying effect of acupuncture on PIPN is mainly derived from studies focusing on peripheral and spinal levels [24]. A recent study from our group identified that electroacupuncture (EA) intervention can effectively alleviate mechanical pain hypersensitivity in model animals through attenuating TLR4-mediated TRPV1 channel up-regulation in DRG neuron [25]. For spinal cord level, it is demonstrated that EA alleviates PIPN in model animals via mechanisms which include the reduction of spinal neuroinflammation and glial cell overactivation, the inhibition of spinal TLR4/NF-κB signaling and phosphorylation of spinal CaMKII as well as activation of opioid and adrenoceptors receptors, etc. [26–29].

Here in this work, considering the importance of peripheral macrophage infiltration to the etiology of PIPN, we aimed to investigate whether EA could possibly interfere with macrophage infiltration in DRG and peripheral nerve to ameliorate symptoms of PIPN model animals. Our results demonstrate that EA can alleviate signs of PIPN in animal model by reducing CCL2/CCR2-mediated pro-inflammatory macrophage infiltration in peripheral sensory ganglia and nerves, without affecting antitumor activity of paclitaxel. Thus, our study highlights the potential application of EA as a non-pharmacological therapy for PIPN. We believe mechanism investigation of EA can further help to facilitate its utilization in clinical management of PIPN.

## Methods and materials

### Animals

C57BL/6 mice (6 weeks, male and female) and BALB/c (6 weeks, female) were used in the present study. Animals were housed in Zhejiang Chinese Medical University Laboratory Animal Facility. The mice were randomly allocated and were housed in cages (5 animals per cage, 12 h dark–light cycle, 24 ± 2 ℃). The cages were standard breeding cages for mice (325 mm × 210 mm × 180 mm). Animals were given free access to water and food. The food was standard mouse chow (protein 18%, fat 5%, and fiber 5%). All animals were allowed 7 days to accommodate the breeding facility ahead of any assay or test.

### Model establishment

**Fig. 1:**
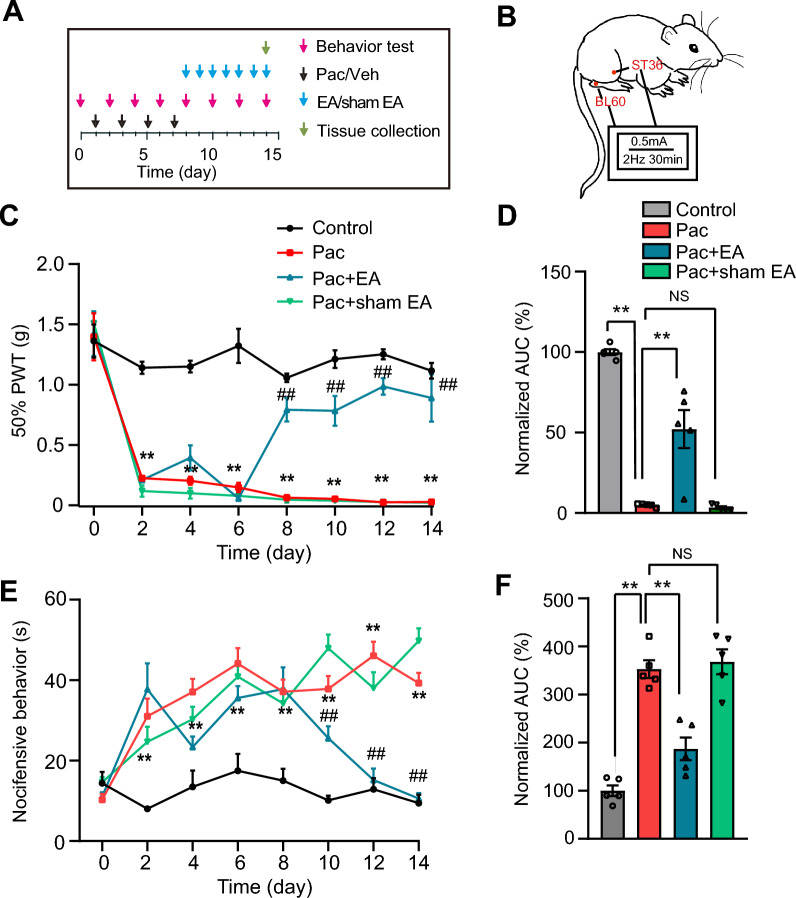
EA intervention alleviates mechanical and cold allodynia of PIPN model male mice. **A** Experimental protocol indicating time points for the establishment of PIPN model in mice, behavior test and EA/sham EA intervention. **B** A schematic description of the locations of ST36 (5 mm laterally of the anterior tibial tubercle) and BL60 (at the level of the ankle, between the tip of the lateral malleolus and the tip of the calcaneus) acupoints in mouse. **C** Effect of repeated 2 Hz EA on 50% PWT of control, Paclitaxel (Pac), Pac + EA and Pac + sham EA groups. ** *p* < 0.01 vs. control group. ^##^ *p* < 0.01 vs. Pac + sham EA group. **D** Normalized area under the curve (AUC) analysis of the curves shown in **C**. ** *p* < 0.01. NS: no significance. **E** Effect of repeated 2 Hz EA intervention on nociceptive behaviors by acetone test of control, Pac, Pac + EA and Pac + sham EA groups. **F** AUC analysis of the curves shown in **E**. *n* = 5 mice/group

The mouse PIPN model was established according to methods as previously described [25, 30]. Briefly, 6 mg/mL of pharmaceutical grade paclitaxel (Hospira Australia Pty. Ltd, Australia) was diluted with sterile 0.9% saline to 1 mg/mL and given at a dosage of 2 mg/kg intraperitoneally (i.p.) in 50 μl/25 g mouse every other day for a total of 4 injections (Day 1, 3, 5 and 7, as shown in Fig. 1A inset). Control animals received an equivalent volume of Cremophor EL (#C875008, Macklin Inc., Shanghai, China) and 95% dehydrated ethanol (1:1) as the vehicle. A successful establishment.

### Mechanical pain hypersensitivity assay

The animals were put in the testing room for 3 days before baseline test to get used to the testing conditions. The mice were individually placed in transparent Plexiglas chambers on an elevated mesh floor for 30 min to acclimate to the testing environment. The mechanical pain hypersensitivities were evaluated by means of the von Frey hairs (UGO Basile, Italy). The von Frey hairs were poked upright to the mid-plantar surface of the hindpaws. Enough force were applied so that the hairs can be slightly bent for 3–5 s according to “Up-Down” testing rules [31, 32]. The 50% paw withdrawal threshold (PWT) was calculated using Dixon test [33]. PWT was evaluated 1 day before PTX injection (Day 0), 1 day after each PTX injection (Day 2, 4, 6 and 8) and every other day after PTX injection (Day 10, 12 and 14) for a consecutive of 8 times (as shown in Fig. 1A inset).

### Cold pain hypersensitivity assay

Cold allodynia was determined by acetone test method as we previously described [34, 35]. The animals were habituated for 30 min as before. Each mouse was sprayed with 20 μl acetone on the plantar surface of the hind paw, and the mouse behaviors within 60 s were recorded using a video recorder. Cold pain hypersensitivities were determined as an accumulation in pain-related behaviors (e.g. licking/shrinking/lifting/flapping) observed after exposure to the acetone. Cold pain hypersensitivity was evaluated 1 day before PTX injection (Day 0), 1 day after each PTX injection (Day 2, 4, 6 and 8) and every other day after PTX injection (Day 10, 12 and 14) for a consecutive of 8 times (as shown in Fig. 1A inset).

### Electroacupuncture (EA) intervention

Based on our previously described EA protocol, we carry out the procedure of EA [25, 36]. Briefly, mice were loosely immobilized, and acupuncture needles of 0.16 mm in diameter were inserted at a depth of 4 mm into bilateral Zusanli (ST36, 5 mm lateral to the anterior tubercule of the tibia) and Kunlun (BL60, at the ankle joint level and between the tip of the external malleolus and calcaneus) acupoints. The needles were connected with HANS acupuncture point nerve stimulator (HANS-200A, Huawei Co., Ltd, Beijing, China). The parameters were set as follows: 2 Hz, square wave current output (pulse width: 0.2 ms), intensities 0.5 mA, time for 30 min. For sham EA intervention, mice were inserted with needles subcutaneously without electric stimulation. EA/sham EA was performed on a daily basis 1 day after the last (the 4th) paclitaxel injection, for a consecutive of 7 times and conducted once daily for 7 consecutive days (as shown in Fig. 1A inset).

### Drug administration

INCB3344 (APExBIO, USA), the CCR2 antagonist was dissolved in dimethyl sulfoxide (DMSO) as stock solution and further diluted to PBS accordingly. INCB3344 (100 µl of 0.17 mM solution/mouse/injection) was administered intravenously into the tail vein on Days 8, 9, and 10 after the first paclitaxel treatment. The dose of INCB3344 was adopted as documented in previous literature [37]. Vehicle group animals were applied 0.1% DMSO in PBS only. Clodronate (Vrije Universiteit Amsterdam, Holland) was used to deplete phagocytic macrophages [38]. Clodronate (1 mg/100 μl per mouse per injection) was intraperitoneally injected on Days 8, 10, and 12 after the first paclitaxel treatment. Mice of vehicle group were administered with liposome only. Recombinant mouse CCL2 (#578402, BioLegend, USA) (0.3 μg/5 μl per mouse per injection) was intrathecally injected on Days 8, 10, and 12 after the first paclitaxel treatment, whereas vehicle group was administered with 0.1% BSA + PBS.

### Real-time quantitative PCR (qPCR)

Total RNA was reversely transcribed to cDNA by means of the PrimeScript RT Reagent Kit. qPCR was performed using TB Green Premix Ex Taq II (Takara Bio Inc., Japan) as master kit with CFX96 Real-Time System (Bio-Rad Laboratories Inc., USA). Each reaction was performed in triplicates and normalized to β-actin gene expression. Cycle threshold (CT) value of each well was deduced with CFX96 Real-Time System software and the average of the triplicates was calculated. ΔΔCT method was utilized to calculate relative quantification [39, 40]. The primer sequence (from 5′ to 3′) is as follows: *b-actin* forward: GTGCTATGTTGCTCTAGACTTCG, reverse: ATGCCACAGGATTCCATACC; *Ccl2* forward: TTAAAAACCTGGATCGGAACCAA, reverse: GCATTAGCTTCAGATTTACGGGT.

### ELISA for CCL2

Mice were sacrificed on Day 14 after EA/sham EA treatment. Bilateral L3-5 DRG were removed and immediately frozen in liquid nitrogen. The ELISA procedure was adopted from our previous studies with minor modifications [41–43]. Tissues were homogenized by means of the Bullet Blender (NextAdvance, USA) in 50 mM Tris base (pH 7.4) and 150 mM NaCl supplemented with protease inhibitor (Roche, Switzerland) and 0.2% Triton X. Homogenization was performed for 20 min at full speed. Then samples were centrifuged at 10,000 × *g* for 10 min at 4 °C. The supernatant was collected and subject to ELISA for CCL2 detection (R&D Systems, USA) according to the manufacture’s instruction. The plate was read at 450 nm using a microplate reader (SpectraMax M4, Molecular Devices, USA). Total protein was determined by BCA assay (Thermo Fisher Scientific, USA).

### Flow cytometry

After EA treatment, mice were sacrificed on Day 14. The L3–5 segments of the DRG were rapidly removed and transferred to digesting solution containing 1 mg/ml collagenase A and 2 mg/ml dispase in DMEM. Then the DRG were incubated at 37 °C with shaking for 1 h at 50 rpm. After removing the supernatant, the cells were washed twice with DMEM and PBS, and filtered through a 70 μm cell filter. 1 × RBC lysis buffer (C3702, Beyotime, China) was used to reduce RBC contamination. Surface expression of F4/80, CD86, CD206 and CD11b was analyzed by flow cytometry. The corresponding antibodies were added to 500 μl PBS containing 5% fetal bovine serum (FBS, Biological Industries, Israel), and incubate in the dark at 4 °C for 1 h. The antibodies used were as follows: rat anti mouse F4/80-PE, BD, #565,410 1:1000; rat anti mouse CD11b-APC, BD, #562,102, 1:1000; rat anti mouse CD86-PE, BD, #560,582, 1:500; rat anti mouse CD206-FITC, BioLegend, 1:500. After staining, the cells were washed in PBS for 3 times. Flow cytometry events were acquired in a CytoFLEX S (Beckman Coulter, USA). Data were analyzed by CytExpert Software (Beckman Coulter, USA).

### Immunofluorescence staining

The procedures for DRG immunofluorescence staining were adopted from our previous studies [36, 44, 45]. Briefly, the mice were sacrificed on Day 7 or 14. The ascending aorta was perfused with 0.9% saline (4 °C) followed by 4% paraformaldehyde in 0.1 M PBS. The L3-5 dorsal root ganglia (DRG) were removed and fixed in 4% paraformaldehyde for 6 h. Then, it is dehydrated in 30% sucrose solution. DRG Sects. (10 µm) were cut on a frozen microtome in longitudinal (Thermo NX50, CA, USA), installed in gelatin-coated glass slides as 8 sets of every 5th serial section, and processed for immunofluorescent staining. After blocking in 5% donkey serum (with 0.3% Triton X-100) in PBS for 1 h at 37 °C and sections were incubated overnight with corresponding primary antibodies an 4 °C. The primary antibodies rabbit anti-CCL2 (#ab7202, abcam), rat anti-F4/80 (#ab6640, abcam), rabbit anti PGP9.5 (#ab108986, abcam), rabbit anti-ATF3 (#HPA001562, Sigma), rabbit anti-NeuN (#ab177487, abcam). After washing in PBS, sections were incubated with corresponding secondary antibodies for 1 h at 37 °C. Sections were viewed by ZEN multifunctional fluorescence microscope (Axio Observer. A1, Zeiss, Germany). Uniformed setting was set for the microscope while capturing images. Three images were randomly selected from each tissue section, averaged, and then analyzed as previously described [46].

### Histopathologic evaluation of the sciatic nerve

Mice were sacrificed on Day 14. The mice were perfused through the ascending aorta with 0.9% saline (4 °C) followed by 2.5% glutaraldehyde in 0.1 M PBS. The sciatic nerve samples were then removed and fixed in 2.5% glutaraldehyde for 1 h, and post-fixed in 1% osmium, 1.5% K_3_[Fe(CN)_6_] and 1% thiocarbohydrazide. Staining was performed overnight with 2% uranium dioxyacetate. These samples were then dehydrated in increasing concentrations of ethanol. Then sample were embedded in paraffin, and sliced. The sciatic nerve sections were prepared and stained with lead citrate and uranyl acetate. These specimens were examined and photographed using a transmission scanning electron microscope (SU8010, HITACHI, Japan) with × 2000 magnification for assessing the myelination integrity and pathological changes. Two non-overlapping and randomly selected observational fields were selected per section. The nerve fibers in these observational fields were evaluated and summarized by an experimenter blinded to the grouping.

### Tumor cell culture and tumor xenograft model establishment

The breast cancer cell line 4 T-1 cells were cultured in DEME/F12 (Gibco, UK) supplemented with 10% FBS (Biological Industries, Israel), 100 units/mL penicillin and 100 μg/mL streptomycin at 37 °C in a humidified atmosphere of 5% CO_2_ and 95% air. For mouse tumor xenograft model, 3 × 10^5^ murine breast cancer 4 T-1 cells were subcutaneously planted into the right sub-axillary fat pad of 6 weeks aged female BALB/c mice. When the size of tumors approaches 100 mm^3^, animals were then assigned to respective groups to get paclitaxel (2 mg/kg, accumulative 8 mg/kg, i.p.) or vehicle treatment for 4 alternative days. Tumor sizes were quantified using the digital caliper. Then the sizes of the tumor were deduced using the following formula: V (mm^3^) = 0.5 × the longest tumor diameter × the shortest tumor diameter^2^ [47].

### In vivo imaging

The mice were injected (i.p.) with fluorescent dye D-fluorescein potassium salt (#2109GR001, BioFRoxx, Germany, 20 mg/kg). Mice were anesthetized with isoflurane, and then placed on the stage of an IVIS Lumina LT in vivo imaging system (PerkinElmer, USA). The fluorescence imaging was performed 10 min post-injection. The luminescence signal intensities were quantified with Living Image software (PerkinElmer, USA).

### Statistical analysis

Statistical analysis was performed with SPSS 19.0. Results were expressed as mean ± SEM. Student’s *t* test was used for comparisons between 2 groups. One-way or two-way ANOVA with repeated measures followed with Tukey’s post hoc test was used for comparison among groups ≥ 3. Comparison was considered significantly different if *p* < 0.05.

## Results

### Electroacupuncture (EA) intervention ameliorates mechanical and cold pain hypersensitivities of a mouse model of paclitaxel-induced peripheral neuropathy

A mouse model of paclitaxel-induced peripheral neuropathy (PIPN) was established according to previous studies [13, 30]. Paclitaxel was administered intraperitoneally (i.p.) at a dose of 8 mg/kg (4 × 2 mg/kg, in every 2 days) (Fig. 1A). Compared with vehicle-treated mice (control group), paclitaxel treatment caused remarkable decrease of 50% paw withdraw threshold (PWT). The mechanical allodynia persisted until the end of the study period (Day 14) (Fig. 1B). Besides, paclitaxel-treated mice exhibited cold allodynia behaviors evaluated by the acetone test, compared with control group (Fig. 1C). The above observations were all in agreement to previous findings, suggesting we have established the PIPN mouse model [30].

Then we began to evaluate the potential effects of EA on pain-related behavior of male PIPN model mice. 2 Hz EA was applied on bilateral ST36 and BL60 acupoints of PIPN model mice, starting on Day 8 every day until Day 14 (Fig. 1A, B). EA intervention improved the mechanical allodynia of PIPN mice. In addition, sham EA intervention did not significantly improved the mechanical allodynia vs. paclitaxel-treated (Pac) group (Fig. 1C). Area under the curve (AUC) analysis further summarized the total action of EA on mechanical allodynia in PIPN model mice through the observation time frame (Fig. 1D). In addition, EA intervention effectively reduced the cold allodynic behaviors of PIPN model mice as well. In contrast, sham EA turned out not to be that effective (Fig. 1E, F).

It is known sex dimorphism is present in chronic pain modulations [48]. These aforementioned data was all obtained from male animals. Therefore, we further evaluated EA’s analgesic effect on female PIPN model mice. Similar to the results obtained from male mice as above, EA significantly ameliorated the mechanical and cold allodynia of female PIPN model mice, whereas sham EA was not effective (Fig. S1A–E). These data suggests EA intervention ameliorates pain hypersensitivities in PIPN model mice of both sexes.

### EA intervention reduced the infiltration of macrophages in DRG and sciatic nerve of paclitaxel-treated mice

**Fig. 2:**
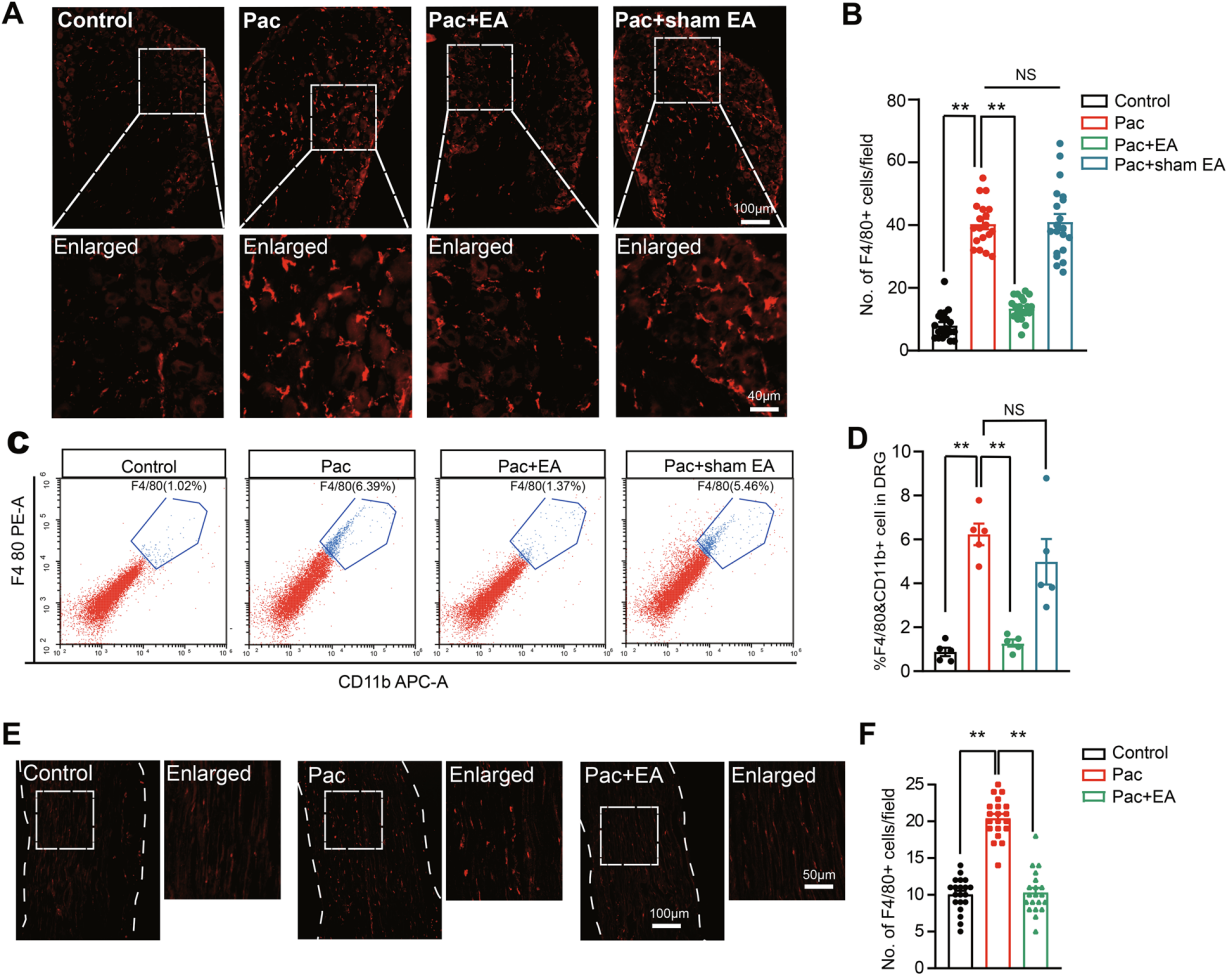
The effect of EA intervention on macrophage infiltrations in DRG of PIPN model mice. **A** Images of immunostaining showing F4/80 staining in DRG. Scale bar indicates 100 μm. **B** Summary of the No. of F4/80 positively stained macrophages per each observation field. 20 sections (pooled from 5 mice/group) were included in each group. **C** Flow cytometry images showing the % of F4/80^+^CD11b^+^ macrophages in DRG. **D** Flow cytometry analysis of the percentage of F4/80^+^CD11b.^+^ cells. *n* = 5 mice/group. **E** F4/80 staining of sciatic nerve of control, Pac, and Pac + EA groups of mice. **F** Summary of the No. of F4/80 positively stained macrophages per each observation field in sciatic nerve as in **E**. 20 sections (pooled from 5 mice/group) were included in each group. ** *p* < 0.01

Studies have shown that macrophages massively infiltrate into DRG in PIPN model animal, which makes a critical contribution to mechanical allodynia of PIPN model animal [14]. Using the macrophage marker F4/80 antibody, our immunofluorescence staining confirmed that number of macrophages (F4/80 positive) was significantly augmented in DRG from paclitaxel-treated mice (Fig. 2A, B). We further confirmed the contribution of infiltrated macrophages to PIPN by in vivo depleting macrophages using clodronate-loaded liposomes (Fig. S2A). Results showed that clodronate administration significantly decreased the number of macrophages in DRG of PIPN model mice (Fig. S2B, C). Furthermore, after macrophage depletion, paclitaxel-treated group of mice showed significantly improved mechanical allodynia (Fig .S2D). Importantly, EA significantly decreased macrophages infiltration induce by paclitaxel treatment on Day 14 (Fig. 2A, B). The immunostaining results were next verified via flow cytometry. Similar to immunostaining results we obtained, flow cytometry experiments revealed a significant increased percentage of CD11b^+^ F4/80^+^ cells in DRG from PIPN model mice on Day 14 (Fig. 2C, D). EA intervention significantly reduced the percentage of CD11b^+^ F4/80^+^ cells in PIPN model mice, whereas sham EA had no obvious effect on Day 14 (Fig. 2C, D). In addition to DRG, our immunostaining further revealed a significant increase in macrophage infiltrations in the sciatic nerve of paclitaxel-treated mice compared with control group on Day 14 (Fig. 2E, F). EA intervention further reduced the macrophage infiltration in sciatic nerves of animals under paclitaxel treatments (Fig. 2E, F).

**Fig. 3:**
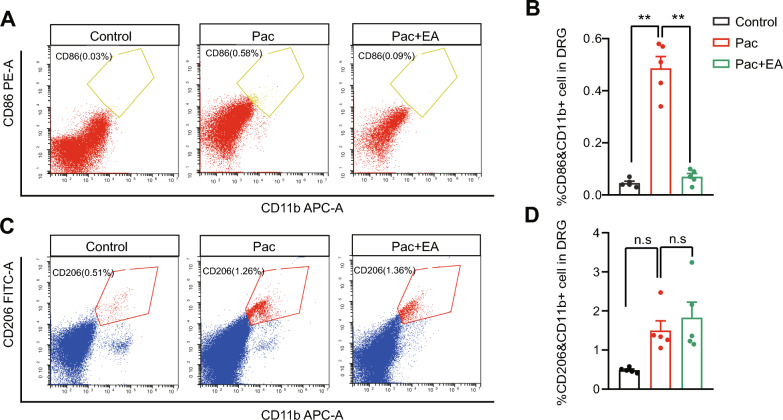
The effects of EA intervention on M1- and M2-like macrophages in DRG of PIPN model mice. **A** Flow cytometry images of % of cells positive for M1-like macrophage markers CD86 and CD11b in DRG of control, Pac, and Pac + EA groups. **B** Analysis of the percentage of CD86^+^CD11b^+^ M1-like macrophages of 3 groups. **C** Flow cytometry images of the % of cells positive for M2-like macrophage marker CD206 and CD11b in DRG of control, Pac, and Pac + EA groups. **D** Analysis of the percentage of CD206^+^CD11b^+^ M2-like macrophages in DRG of 3 groups. ** *p* < 0.01. NS: no significance. *n* = 5 mice/group

Activated macrophages can be sub-categorized into two major groups, M1- or M2-like macrophages. M1-like macrophages are predominantly involved in pro-inflammatory processes, whereas M2-like macrophages predominantly exert anti-inflammatory effects [49]. Therefore, we proceeded to examine on which specific groups of macrophages EA may produce its interventional effects via flow cytometry method. Flow cytometry experiments revealed that the proportion of CD11b^+^CD86^+^ (M1-like) cells in all cell populations was increased in DRG of paclitaxel-treated group of mice and EA intervention significantly reduced the increased proportion of CD11b^+^CD86^+^ cells by paclitaxel treatment on Day 14 (Fig. 3A, B). In addition, flow cytometry also detected a proportion of CD11b^+^CD206^+^ (M2-like) cells in all cell populations in DRG of paclitaxel-treated group of mice (Fig. 3C, D). However, EA intervention did not exhibits any significant effects on the proportion of CD11b^+^CD206^+^ cells in DRG by paclitaxel treatment on Day 14 (Fig. 3C, D). Thus, these results in all indicate that EA intervention can significantly reduce the infiltration of macrophages, including M1-like pro-inflammatory macrophages, in DRG of paclitaxel-treated mice.

### EA intervention ameliorates macrophage infiltration and mechanical allodynia of PIPN model mice by affecting CCL2/CCR2 signaling in DRG

Former work has already demonstrated the potent macrophage chemoattractant CCL2 was remarkably increased in DRG neurons of model rats of PIPN, and neutralization of CCL2 can ameliorate macrophage infiltration in DRG and reduces pain hypersensitivities in PIPN model rats [13, 17]. These findings demonstrate a critical contribution of CCL2 signaling in mediating macrophage infiltration and PIPN in rats. Therefore, we decided to focus on CCL2 signaling in our following studies. Since the above mentioned findings were all obtained from rats, we next started by testing whether CCL2 expression was up-regulated in DRG neurons of the mouse model of PIPN we currently used.

Bilateral DRG (L3-L5) of mouse were collected on Day 14 as shown in Fig. S3A. qPCR experiments showed that paclitaxel treatment induced the overexpression of *Ccl2* gene in mouse DRG on Day 14 (Fig. S3A). Immunostaining indicated CCL2 protein expressions were obviously up-regulated in the DRG from PIPN mouse model vs. control on Day 14 (Fig. S3B and C). Immunostaining also identified that CCL2 was mainly co-localized with the neuron marker NeuN, but not the glial marker GFAP, suggesting CCL2 was exclusively produced from DRG neurons in PIPN model mice (Fig. S3D). We further explored whether CCL2-related signaling mediated macrophage infiltration in PIPN model mice. CCL2 exerts potent chemotaxis effect on macrophages via receptor CCR2 [16]. Therefore, INCB3344, as specific blocker for CCR2, was intravenously injected (0.17 mM/100 μL, i.v.) at Day 8, 9 and 10 time points after PIPN model establishment to pharmacologically block CCR2 function (Fig. S3E). Immunostaining showed that INCB3344 treatment significantly decreased the infiltrated F4/80^+^ macrophages caused by paclitaxel compared with vehicle treatment (Fig. S3F–H). Furthermore, INCB3344 remarkably reduced the mechanical allodynia of PIPN model (Fig. S3I). Thus, these results indicate that CCL2/CCR2 signaling mediates the macrophage infiltration in DRG, which results in mechanical allodynia of PIPN model mice.

**Fig. 4:**
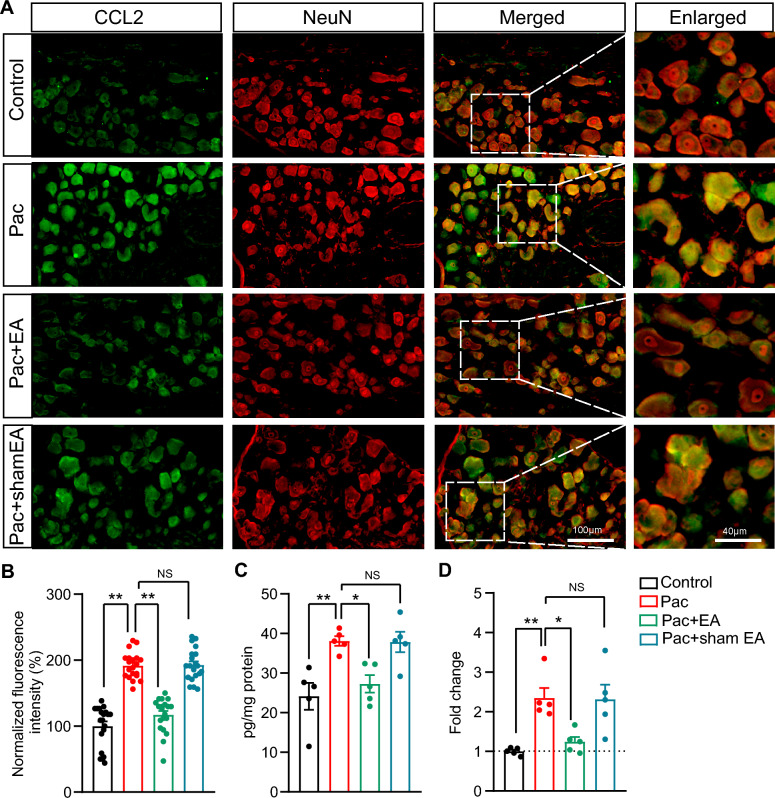
EA intervention reduced the overexpression of CCL2 in DRG of PIPN model mice. **A** CCL2 antibody staining (in green) of DRG from 4 groups of mice. The neuron marker NeuN is in red. **B** Summary of the normalized fluorescence intensity (%) of CCL2 staining in DRG. The value derived from the control was taken as 100%, and all other group values were normalized. 20 sections (pooled from 5 mice/group) were included in each group. **C** ELISA showing the level of CCL2 protein in DRG lysates of each group. **D** qPCR results showing *Ccl2* gene expression in DRG of each group. *n* = 5 mice/group. ** *p* < 0.01, * *p* < 0.05. NS: no significance. Scale bar indicates 100 μm

Based upon these results obtained above, we next proceeded to examine whether EA may reduce macrophage infiltration through affecting CCL2/CCR2 signaling in DRG of PIPN model mice. Bilateral L3-L5 DRG were collected on Day 14 as shown in. Figure 1A. Immunostaining revealed EA intervention significantly decreased CCL2 overexpression in DRG neurons from PIPN model mice on Day 14 (Fig. 4A, B). Sham EA does not have similar effects (Fig. 4A, B). We then quantified CCL2 protein expression by means of ELISA assay. As shown in Fig. 4C, ELISA results confirmed that CCL2 protein expression was increased in DRG of PIPN model mice on Day 14. ELISA further confirmed EA intervention could significantly reduce the overexpression of CCL2, whereas sham EA had no obvious effect (Fig. 4C). We then tested whether the modulatory effect of EA on CCL2 protein expression is due to modulation on the gene expression. qPCR indicated EA intervention was capable of reducing *Ccl2* gene expression upregulation in DRG of PIPN model mice on Day 14, whereas sham EA had no obvious effect (Fig. 4D).

**Fig. 5:**
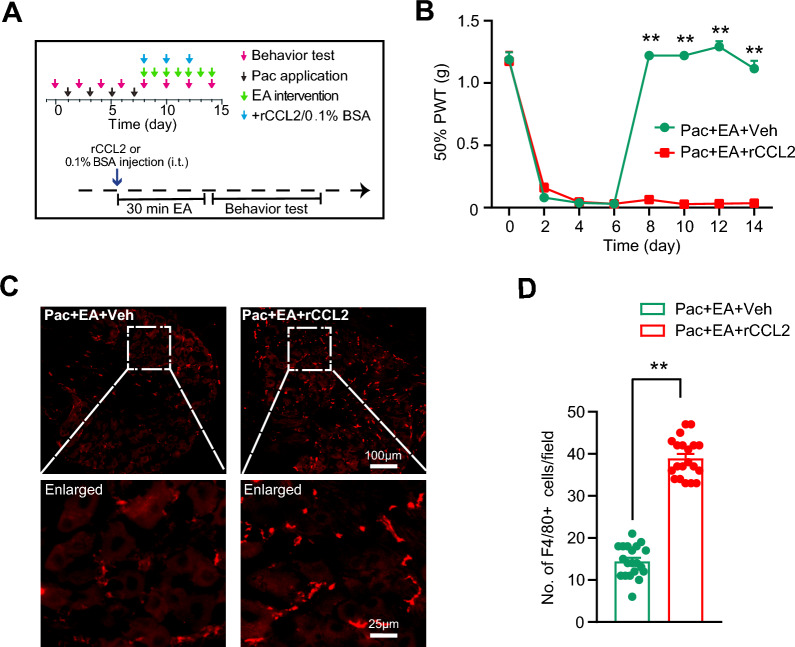
Application of recombinant mouse CCL2 reversed the anti-allodynia effect of EA on PIPN model mice. **A** Time schedule for PIPN model, behavior test, EA intervention and recombinant mouse CCL2 (rmCCL2) protein application. rmCCL2 was intrathecally administered (0.3 μg/5 μl/mouse). 0.1% BSA dissolved in PBS was used as vehicle (Veh). **B** Change in 50% PWT of Pac + EA + Veh and Pac + EA + rmCCL2 groups of mice. *n* = 5 mice/group. ** *p* < 0.01 vs. Pac + EA + rmCCL2 group. **C** F4/80 staining of macrophages in DRG. **D** Summary of the No. of F4/80^+^ macrophages in two groups as in **C**. 20 sections (pooled from 5 mice/group) were included in each group. ** *p* < 0.01. Scale bar indicates 100 μm

We then utilized a recombinant mouse CCL2 protein (rmCCL2) to examine its potential effects on EA-induced analgesia in PIPN mice. rmCCL2 (0.3 μg/5 μl/mouse) or corresponding vehicle (0.1% BSA + PBS) was administered intrathecally (i.t.) to PIPN mice 30 min ahead of EA intervention at Day 8, 10 and 12 time points after PIPN model establishment (Fig. 5A). We found that exogenously administration of excessive rmCCL2 could remarkably reverse the anti-allodynia action of EA on PIPN mice at these time points (Fig. 5B). Immunostaining further revealed that the exogenously applied rmCCL2 could significantly increase the number of F4/80^+^ macrophages infiltration in DRG of EA-treated PIPN model mice (Pac + EA + rCCL2) compared with vehicle-administered mice (Pac + EA + Veh) (Fig. 5C, D). This finding demonstrates that exogenously administered rmCCL2 can counteract EA-induced anti-allodynic effect on PIPN model mice by promoting macrophage infiltrations in DRG.

### EA intervention ameliorates neuronal injury in DRG and improves pathological changes in sciatic nerve and loss in intra-epidermal nerve fibers of PIPN model mice

**Fig. 6:**
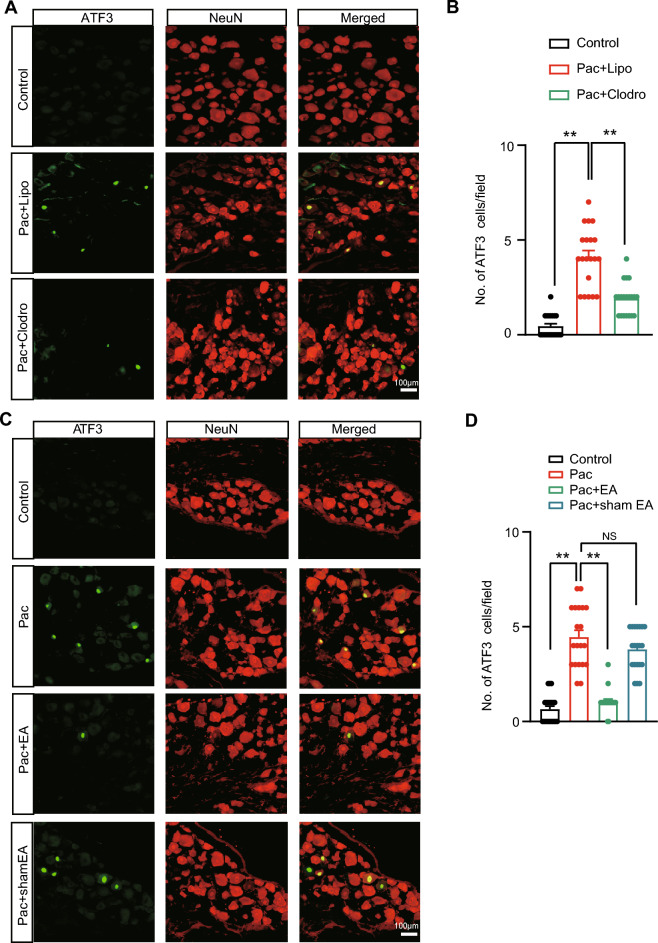
EA intervention ameliorates neuronal damage in DRG of PIPN model mice. **A** Representative immunofluorescence images showing ATF3 expression (in green) in DRG of control and PIPN model mice which received liposome (vehicle) or clodronate to deplete macrophages. NeuN (in red) was used to mark DRG neurons. Bilateral L3-L5 DRG were collected on Day 14 for each group. **B** Summary of the No. of ATF3 positively stained (ATF3^+^) cells in these 3 groups. **C** ATF3 expression in DRG of PIPN model mice after EA or sham EA intervention. **D** Summary of the No. of ATF3^+^ cells in 4 groups as indicated. ** *p* < 0.01. Scale bar indicates 100 μm. 20 sections (pooled from 5 mice/group) were included in each group

It is known that paclitaxel treatment can cause remarkable neuronal injury in DRG as manifested by an obvious increase in neuronal damage marker-activated transcription factor 3 (ATF3) positively stained neurons [30]. However, it is still not known yet whether the neuronal injury caused by paclitaxel treatment in DRG was due to paclitaxel-induced macrophage infiltration. DRG was collected on Day 14. We first confirmed ATF3 positive stained DRG neurons were increased in paclitaxel-treated group of mice via immunostaining on Day 14 (Fig. 6A, B). We further showed that the depletion of macrophages via clodronate-loaded liposomes significantly reduced amount of ATF3^+^ neurons induced by paclitaxel (Fig. 6A, B). Thus, this results clearly indicates an important contribution of macrophage infiltrations to paclitaxel-induced neuronal injury in the DRG. Since EA can ameliorate macrophage infiltration in DRG caused by paclitaxel treatment, our group proceeded to explore the effects of EA on paclitaxel-triggered neuronal damage. Immunostaining showed EA intervention could reduce the amount of ATF3^+^ DRG neurons induce by paclitaxel treatment on Day 14 (Fig. 6C, D).

**Fig. 7:**
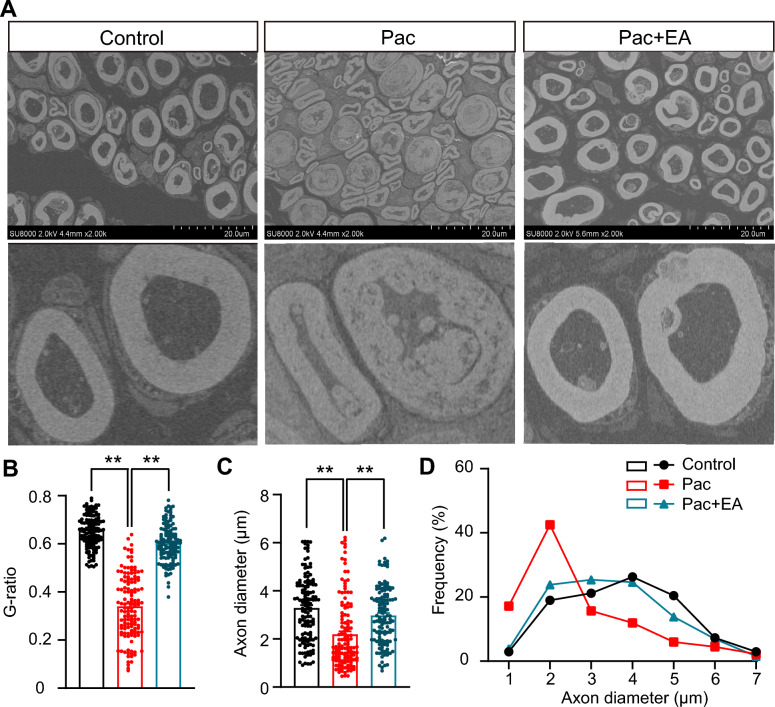
EA intervention improves the impairments in sciatic nerve morphology of PIPN model mice. **A** Representative transmission electron micrographs showing the sciatic nerve in Control, Pac, and Pac + EA groups of mice. Sciatic nerve tissues were collected on Day 14 for each group. Scale bar: 20 μm. **B** G-ratio summary of myelinated axons of 3 groups. **C** Quantitative results showing axonal diameters of 3 groups. **D** Size distribution histogram of axon diameter. 137, 134, 130 axons (pooled from 3 mice/group) were included in Control, Pac, and Pac + EA group, respectively. ** *p* < 0.01

Electro-microscopy identified obvious degenerative changes occurred in the sciatic nerve of PIPN model mice on Day 14, which included an aberrant thickening and folding of myelin sheath and axonal shrinkage (Fig. 7A). We found that the averaged axonal diameter of PIPN group became smaller than control group. EA intervention significantly reversed this change in axonal diameter caused by paclitaxel treatment (Fig. 7A, B). By analyzing G-ratio (axon diameter divided by fiber diameter) of the nerve fiber, we found that G-ratio were significantly decreased by paclitaxel treatment, whereas EA intervention significantly reversed the change in G ration (Fig. 7A, C). We then analyzed the distribution of myelinated axons according to their diameters. Summarized results showed that paclitaxel treatment could remarkably shifted the distribution leftwards (namely to smaller-diameter). EA intervention could reverse the leftward shift in axonal diameter by paclitaxel treatment (Fig. 7D).

**Fig. 8:**
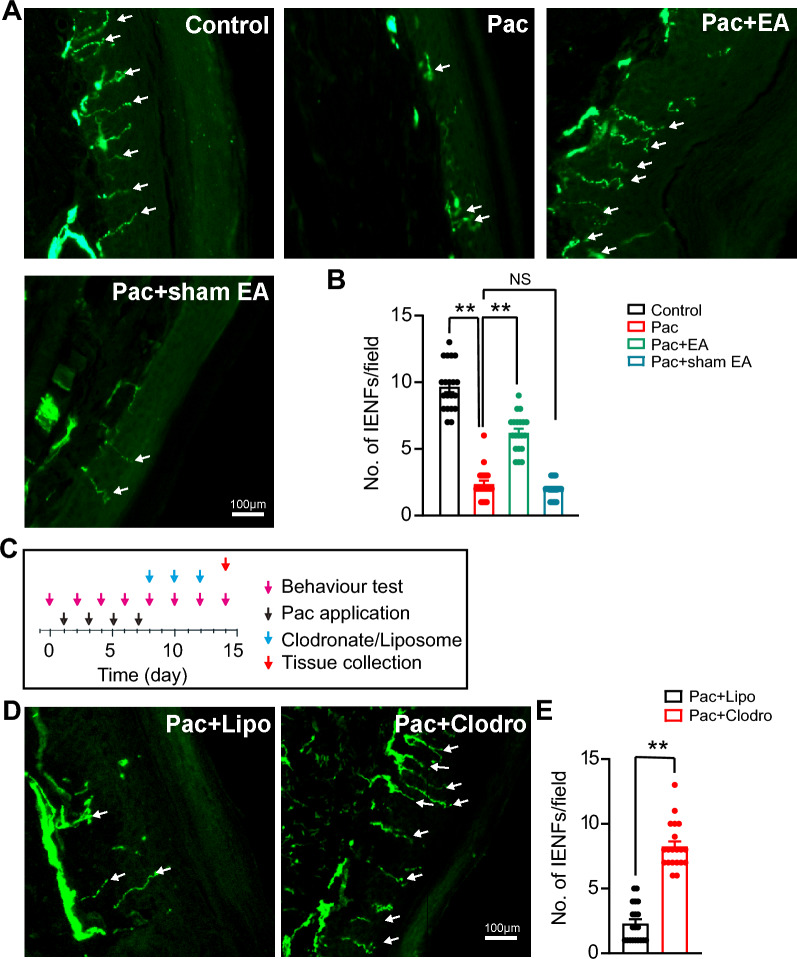
Depleting macrophages or EA intervention can improve the loss of intra-epidermal nerve fibers in PIPN model mice. **A** PGP9.5 antibody staining of the intra-epidermal nerve fibers (IENFs) from footpad of 4 groups mice. Footpad tissues were collected on Day 14 for each group. **B** Summary of the No. of IENFs labeled with PGP9.5 from 4 groups as in **A**. 20 sections (pooled from 5 mice/group) were included in each group. **C** Schematic picture showing time points for the experiments and tissue collection as performed in **D**. **D** Representative immunofluorescence images indicating IENFs from Pac + Lipo, and Pac + Clodro groups. **E** Summary of the No. of IENFs labeled with PGP9.5 from the two groups as in **D**. 20 sections (pooled from 5 mice/group) were included in each group. ** *p* < 0.01. Scale bar: 100 μm

It has been known that paclitaxel can trigger significant losses in intra-epidermal nerve fibers (IENFs) of rats, which is considered as an important mechanism leading to peripheral neuropathy [50]. In this study, we continued to monitor the IENFs in the mouse hind paw by PGP9.5 immunostaining. The time points for the treatment and tissue collection were shown in Fig. 1A. We first confirmed that, on time point of Day 14, paclitaxel could cause a remarkable decrease in the number of PGP9.5 positively stained IENFs in mouse model of PIPN. We further identified EA intervention obviously restored the loss of IENFs caused by paclitaxel treatment (Fig. 8A, B). Sham EA has no obvious effects (Fig. 8A, B). Similarly, depleting macrophages using clodronate significantly ameliorated the loss in IENFs in PIPN model mice on Day 14 (Fig. 8C, E), confirming a critical contribution of macrophage infiltration to the loss in IENFs by paclitaxel treatment as previously reported [13]. Therefore, these above results demonstrate that EA can ameliorate neuronal injury in DRG, improves pathological impairments in sciatic nerve and restore the loss in IENFs in PIPN model mice via attenuating macrophage infiltration.

### EA intervention does not affect antitumor activity exerted by paclitaxel treatment

**Fig. 9:**
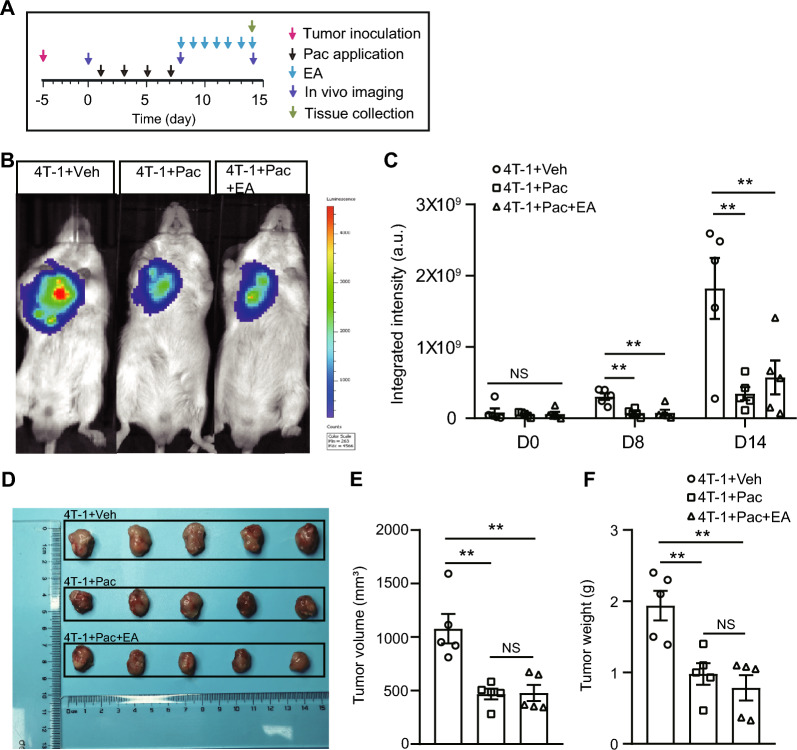
EA intervention does not affect the antitumor activity of paclitaxel on mice inoculated with breast cancer cells. **A** Illustrated time points for the establishment of PIPN in mice, EA intervention, in vivo imaging, tumor cell inoculation and tissue collection. **B** Representative in vivo imaging showing murine breast cancer expansion using D-fluorescein in 4 T-1 + Vehicle, 4 T-1 + Pac (Paclitaxel) and 4 T-1 + Pac + EA group on Day 14. **C** Summarized integrated intensity of D-fluorescein imaging on Day 0, 8 and 14. **D** Pictures showing autopsies of the tumors of 3 groups. **E** Quantitative results showing tumor volume. **F** Quantitative results showing tumor weight. ** *p* < 0.01. *n* = 5 mice/group

Lastly, we evaluated whether EA intervention might affect the antitumor activity of paclitaxel. To address this issue, 3 × 10^5^ murine breast cancer 4 T-1 cells were subcutaneously implanted to the subaxillary fat pad of 6 weeks old female BALB/c mice. As the size of the tumor approaches 100 mm^3^, the mice were then assigned to get paclitaxel, paclitaxel + EA, or vehicle treatment at time points as indicated in Fig. 9A. We then performed a noninvasive in vivo imaging of the murine breast cancer using the fluorescent dye D-fluorescein potassium salt. 4 T-1 + Veh group mice showed remarkable increase in the chemiluminescent intensity at Day 8 and 14. In contrast, the group of mice receiving paclitaxel treatment (4 T-1 + Pac) showed significantly reduced chemiluminescent intensity compared with 4 T-1 + Veh group mice, demonstrating the antitumor activity of paclitaxel. Moreover, the group of mice receiving paclitaxel plus EA treatment (4 T-1 + Pac + EA) also showed significantly reduced chemiluminescent intensity compared with 4 T-1 + Veh group mice and showed no significance compared with 4 T-1 + Pac group (Fig. 9B, C). At Day 14, the animals were sacrificed and the tumors from each group were collected and then measured thereafter. Paclitaxel significantly reduced both of the tumor volume and weight (Fig. 9D, F). Meanwhile, EA intervention did not affect the antitumor effects of paclitaxel (Fig. 9D, F). These findings demonstrate that applying EA to counteract paclitaxel-induced peripheral neuropathy does not affect the antitumor activity of paclitaxel.

## Discussion

Here, our work shows that EA intervention can exert ameliorative effect on mechanical and cold pain hypersensitivities as well as reduce the neuronal damage and loss in intra-epidermal nerve fibers in PIPN model mice. We observed a remarkable macrophage infiltration in both DRG and sciatic nerve of PIPN model mice, which were significantly reduced by EA intervention. Flow cytometry further revealed that EA mainly affected M1-like pro-inflammatory macrophage infiltration, whereas it had no obvious effect on M2-like macrophages. DRG neurons released the chemoattractant CCL2 that acted on macrophage CCR2 to recruit macrophage infiltration in PIPN model mice. EA intervention significantly reduced CCL2 production by DRG neurons and thus reduced macrophage infiltration. Furthermore, EA intervention reduced neuronal damage, sciatic nerve impairments and restored the loss in IENFs in PIPN model mice. Lastly, in mice that were inoculated with breast cancer cells, EA intervention did not affect paclitaxel-induced antitumor effect. Therefore, these findings in all suggest that EA intervention can alleviate signs of PIPN by reducing pro-inflammatory macrophage infiltration in peripheral sensory system. Thus, our study highlights that acupuncture may serve as a convenient and non-pharmacological therapy for alleviating PIPN in patients undergoing chemotherapy with paclitaxel.

Upon nerve injury or neuron damage, macrophage can infiltrate into peripheral sensory ganglia or peripheral sensory nerve to produce a number of pro-inflammatory cytokines to initiate and maintain neuropathic pain via neuro-immune crosstalk [9]. It is known that there is a massive macrophage infiltration in DRG of PIPN model animals. Pharmacologically depleting macrophages can attenuate the pain hypersensitivity in PIPN model animals. Depleting macrophages significantly relieved pain hypersensitivities, as well as the neuronal apoptosis and loss in intra-epidermal nerve fibers of PIPN model rats, demonstrating a crucial contribution of infiltrating macrophages to the etiology of PIPN [13, 15]. Here in this study, we observed similar macrophage infiltration in DRG as well as sciatic nerve of PIPN model mice. We further observed that EA intervention can significantly attenuate macrophage infiltration in both DRG and sciatic nerve of PIPN model mice. This effect of EA on macrophage infiltration may constitute a major mechanism underlying its therapeutic effect on PIPN.

Activated macrophages can be sub-categorized into two major groups, M1- or M2-like macrophages. M1-like macrophages are predominantly involved in pro-inflammatory processes, whereas M2-like macrophages predominantly exert anti-inflammatory effects. M1-like macrophages can produce reactive oxygen species (ROS) and pro-inflammatory cytokines or mediators that can trigger activation or sensitization of the sensory neurons, contributing to pain [9, 51]. But it still remains uninvestigated which type of macrophages were increased in DRG of PIPN model animals until now. Here, we found that the percentage of M1-like macrophages were significantly increased by paclitaxel treatment, whereas M2-like macrophages were not. This result suggests that M1-like macrophages may make major contribution to the pain hypersensitivity and etiology of PIPN. Based upon this observation, we continued to check which subtypes of macrophages EA may affect. We found that EA could predominantly attenuate the infiltration of M1-like pro-inflammatory macrophages but barely had any effect on M2-like anti-inflammatory macrophages. Hence, our work provides compelling evidence to show that EA is capable of reducing M1-like macrophage infiltration in DRG of PIPN model mice. In addition to our findings, some other studies also find that EA can reduce infiltration or activation of M1-like macrophages in the inflamed paw of rheumatoid arthritis or colon tissue of acute colitis model animals [52, 53]. Therefore, these findings in all suggest that EA intervention is capable of modulating M1-like pro-inflammatory macrophages in inflammation or nerve injury condition to exert therapeutic effect.

Macrophages can be recruited by chemokines, including CCL2, CXCL1, CXCL2, etc. Previous studies identified that the potent macrophage chemoattractant CCL2 immunostaining is significantly increased in DRG neurons of paclitaxel-treated animals. Applying neutralizing antibody against CCL2 reduced the macrophage infiltrations in DRG of paclitaxel-treated animals [13]. Here our work confirmed the significant up-regulation of CCL2 expression in DRG neurons of PIPN model mice. Our work further found that pharmacologically blocking CCL2 receptor CCR2 results in reduced macrophage infiltration. These findings in all suggest DRG neuron-produced CCL2 induces macrophage infiltration in PIPN model animals via receptor CCR2. Based upon these findings, our work continued to check the effect of EA intervention on CCL2 in PIPN model mice. Our results showed that EA obviously reduced CCL2 overexpression in DRG neurons of PIPN model mice. Moreover, exogenously applying rmCCL2 could markedly reverse the anti-allodynic effects of EA intervention on PIPN model mice and increase the number of F4/80^+^ macrophages infiltration in DRG of EA-treated PIPN model mice. Therefore, these results suggest that EA may affect CCL2 overexpression in DRG neurons to reduce macrophage infiltration and produce anti-allodynic effect on PIPN model mice.

However, it should be noted that some additional chemokines or signaling molecules may also contribute to macrophage infiltration mechanism besides the aforementioned chemokine CCL2. In a recent study, it was reported that β-galactoside binding protein galectin-3, which was produced from Schwann cells, contributes to macrophages infiltration in the sciatic nerve and mechanical hypersensitivity in PIPN model animals [54]. The chemokine CX3CL1 has been reported to be upregulated in DRG neurons and blocking CX3CL1 reduces macrophage infiltration, neuron apoptosis and alleviates mechanical allodynia in PIPN model animals [15]. Our recent work also indicates that IL-33/ST2 signaling can trigger macrophage infiltration in the incised skin of a mouse model of incisional pain [51]. It is well established that EA exerts its therapeutic effects via acting on multiple targets or signaling pathways. Therefore, it remains likely that EA may also intervene with other participating signaling molecules or pathways involved in macrophage infiltration as mentioned above to reduce macrophage infiltration in DRG and relieve pain of PIPN. Thus, it will be tempting to continue to explore these potential mechanisms underlying EA-induced ameliorating effect on PIPN in future studies.

Here, in addition to pain hypersensitivities, we also studied the therapeutic effects of EA on other signs accompanying PIPN. Our result indicated that EA intervention produce effective protection to the damages of peripheral nerves upon paclitaxel treatment. Previous work demonstrated paclitaxel-treated animals showed a loss in IENFs in hind paws [13]. Depleting macrophages can restore the loss in IENFs, suggesting macrophages make important contribution to this pathological changes in peripheral nerves [13]. The loss in IENFs has been shown to be correlated with the progression of paclitaxel-induced mechanical hypersensitivity [50]. Furthermore, paclitaxel can cause significant neuronal damage as indicated by ATF3 [15]. In this study, we further found that the neuronal damage can be reduced by depleting macrophages, suggesting macrophages contributes to neuronal damage. Our study found that EA can significantly improve the lost in IENFs and reduce ATF3 overexpression, achieving similar effect with pharmacologically depleting macrophages with clodronate. Furthermore, electro-microscope reveals dramatic morphological changes in sciatic nerve of mice receiving paclitaxel, whereas EA could improve these morphological changes. Thus, these findings indicate that EA is able to improve these typical features occurred in DRG and peripheral nerves of PIPN model mice. The corresponding mechanisms may involve the modulation of macrophage infiltration in DRG and the peripheral nerve.

CIPN remains a challenging clinical condition for physicians. It is difficult for patients to get satisfactory treatment since effective therapeutic options available for CIPN are very limited [6]. The only drug that has gained enough evidence to support the usage for established painful CIPN is duloxetine, according to guideline from American Society of Clinical Oncology (ASCO) [6]. However, the amount of benefit from duloxetine is limited and unwanted side effects may occur [6, 55]. Therefore, exploring potential alternative methods for CIPN is of huge clinical importance. Recently, a number of clinical trials demonstrate the therapeutic effects of acupuncture on pain in patients undergoing chemotherapy using paclitaxel, with well tolerance and safety among patients [22, 23]. But it should be noted that these clinical trials are still at preliminary stage and report that acupuncture only reduces the incidence of patients to develop high grade PIPN during chemotherapy. A larger scale, randomized controlled trial of acupuncture vs. placebo is needed to determine whether acupuncture is effective in ameliorating the severity of PIPN. Here, our work demonstrates that EA has the capability of ameliorating painful signs of PIPN in model animals and further explores the mechanism that underlying EA’s effect. Therefore, our work, together with others, suggest that EA could be a promising therapeutic option for PIPN. Anyhow, further studies will be necessary to comprehensively evaluate the therapeutic potentials of EA on patients with PIPN, with the focus on EA’s effect, optimal stimulating parameters, acupoints selection, treatment safety and patients’ tolerance, etc.

## Conclusions

Our study reveals that EA can alleviate signs of PIPN in model animals by reducing pro-inflammatory macrophage infiltration in peripheral sensory ganglia and nerves. Furthermore, EA intervention does not affect the antitumor activity of paclitaxel. Our study supports EA can be potentially used as a non-pharmacological therapy for PIPN management. Future clinical studies will be needed to systematically explore and evaluate therapeutic potentials of EA on PIPN patients.

## Declaration

## Supplementary Information

Additional file1 (DOCX 124 KB)

Additional file2 (DOCX 167 KB)

Additional file3 (DOCX 469 KB)

## Data Availability

The key data are contained in the manuscript. Further request can be obtained from the corresponding author Dr. Boyi Liu.
