# Supplementary material for: Electroacupuncture alleviates paclitaxel-induced peripheral neuropathy by reducing CCL2-mediated macrophage infiltration in sensory ganglia and sciatic nerve
Source: Chin Med. 2025 Jan 13;20:9. doi: 10.1186/s13020-024-01023-8 (PMC11727193; doi:10.1186/s13020-024-01023-8)
Supplement: Supplementary file 1 — Additional file1 (DOCX 124 KB) [file 13020_2024_1023_MOESM1_ESM.docx]

**Fig. S1 EA intervention alleviates mechanical and cold allodynia of female PIPN model mice.** (A) Experimental protocol indicating time points for the establishment of PIPN model in female mice, behavior test and EA/sham EA intervention. Please be noted that female mice were used for this experiment. (B) Effect of repeated 2 Hz EA on 50% PWT of control, Paclitaxel (Pac), Pac + EA and Pac + sham EA groups of female mice. ^**^*p*<0.01 vs. control group. ^##^*p*<0.01 vs. Pac + sham EA group. (C) Normalized area under the curve (AUC) analysis of the curves shown in panel B. n = 6 mice/group. ^**^*p*<0.01. (D) Effect of repeated 2 Hz EA intervention on nocifensive behaviors caused by acetone test of control, Pac, Pac + EA and Pac + sham EA groups of female mice. (E) AUC analysis of the curves shown in panel D. n = 5 mice/group.
