# Supplementary material for: Electroacupuncture alleviates paclitaxel-induced peripheral neuropathy by reducing CCL2-mediated macrophage infiltration in sensory ganglia and sciatic nerve
Source: Chin Med. 2025 Jan 13;20:9. doi: 10.1186/s13020-024-01023-8 (PMC11727193; doi:10.1186/s13020-024-01023-8)
Supplement: Supplementary file 2 — Additional file2 (DOCX 167 KB) [file 13020_2024_1023_MOESM2_ESM.docx]

**Fig. S2 Depleting macrophages ameliorates mechanical** **allodynia of PIPN model mice.** (A) Experimental protocol. Clodronate (Clodro) was injected (i.v.) to deplete macrophages. Liposome was used as vehicle control. (B) Representative immunofluorescence images indicating macrophage antibody (F4/80) staining of DRG from the Pac+ Lipo, Pac+ Clodro group of mice. (C) Summary of the number of F4/80^+^ macrophages of the two groups as in panel B. 20 sections (pooled from 5 mice/group) were included in each group. (D) Effect of clodronate/liposome administration on 50% PWT of two groups of mice. ^**^*p*<0.01. n = 5 mice/group. Scale bar indicates 100 μm.
