# Supplementary material for: Electroacupuncture alleviates paclitaxel-induced peripheral neuropathy by reducing CCL2-mediated macrophage infiltration in sensory ganglia and sciatic nerve
Source: Chin Med. 2025 Jan 13;20:9. doi: 10.1186/s13020-024-01023-8 (PMC11727193; doi:10.1186/s13020-024-01023-8)
Supplement: Supplementary file 3 — Additional file3 (DOCX 469 KB) [file 13020_2024_1023_MOESM3_ESM.docx]

**Fig. S3 CCL2 is overexpressed in DRG neurons of PIPN model mice and contributes to macrophage infiltration and mechanical allodynia.** (A) The expression of *Ccl2* gene by qPCR assays. DRG were collected on Day 14. (B) Representative immunofluorescence images indicating CCL2 antibody staining of DRG from the control and Pac group. (C) Summary of the normalized fluorescence intensity (%) of CCL2 immunostaining in DRG on Day 14. (D) Representative immunofluorescence images indicating CCL2 exclusively co-localized with the neuronal marker NeuN (top) but barely with the astrocytic marker GFAP (bottom). (E) Experimental protocol for CCR2 antagonist INCB3344 administration. (F&G) Representative immunofluorescence images indicating F4/80^+^ macrophages in DRG from the Pac + PBS (F) and Pac + INCB (G) group. 20 sections (pooled from 5 mice/group) were included in each group. DRG were collected on Day 14 as shown in panel E. (H) Summary of the number of F4/80^+^ macrophages of the two groups. (I) Effect of INCB3344/PBS administration on 50% PWT of Pac + PBS and Pac + INCB group of mice. n = 5 mice/group.^**^*p*<0.01. Scale bar indicates 100 μm.
